# Supplementary material for: Point-of-care ultrasound of the inferior vena cava for intravascular volume assessment during intravenous albumin infusion in patients with cirrhosis
Source: JHEP Rep. 2025 Aug 20;7(11):101559. doi: 10.1016/j.jhepr.2025.101559 (PMC12529371; doi:10.1016/j.jhepr.2025.101559)
Supplement: Multimedia component 1 [file mmc1.pdf]

Point-of-care ultrasound of the inferior vena cava for intravascular volume assessment during intravenous albumin infusion in patients with cirrhosis

Daniel Segna, Fabio Brazerol, Pompilia Radu, Gerard Angeles Fite, Jaime Bosch, Annalisa Berzigotti

Table of contents

Supplementary methods……………………………………………………………….2

Fig. S1……………………………………………………………………………………...3

Table S1……………………………………………………………………………………4

Table S2……………………………………………………………………………………5

Stratified analyses for changes in IVC^{max}, IVC^{min}, and IVCCI after PLR and IV albumin infusion

All measurements (n=81)

There were significant inter-sex differences with a significantly higher IVC^{min} and lower IVCCI after IV albumin in female vs. male participants (p=0.01 vs p= 0.02, respectively), whereas IVC^{max} was comparable between these two groups. In patients with more than 5 liters ascites removed and therefore undergoing large volume paracentesis (LVP), both IVC^{min} and IVC^{max} were significantly lower before PLR, whereas IVC^{min} was significantly lower and IVCCI significantly higher at PLR, compared to patients < 5 liters ascites removed. This effect was no longer present before and after IV albumin infusion. There were no significant differences between groups stratifying for Child Pugh Turcotte score (B vs C) at any time point (Table 3).

Individual patients with data from first inclusion (n=55)

In a sensitivity analysis only using data from the first inclusion of every patient, only IVCCI remained significantly lower in women than in men after IV albumin (21.8% vs 30.2%, p=0.04).In patients with LVP, IVC^{min} was significantly decreased (p=0.03) and IVCCI increased (p<0.01) at PLR, whereas IVC^{min} and IVCCI were significantly lower at PLR, compared to patients < 5 liters ascites removed. On the other hand, solely IVC^{max} was significantly higher in patients with LVP after IV albumin (22.5 vs 19.4 cm, p<0.01). When stratifying for Child stage, only IVC^{max} before PLR was significantly higher in Child C than B (15.7 vs. 13.1 cm, p=0.03) while all other parameters were not different in these two groups at any time point.

Fig. S1: Distribution of sodium (mmol) before IV albumin at baseline

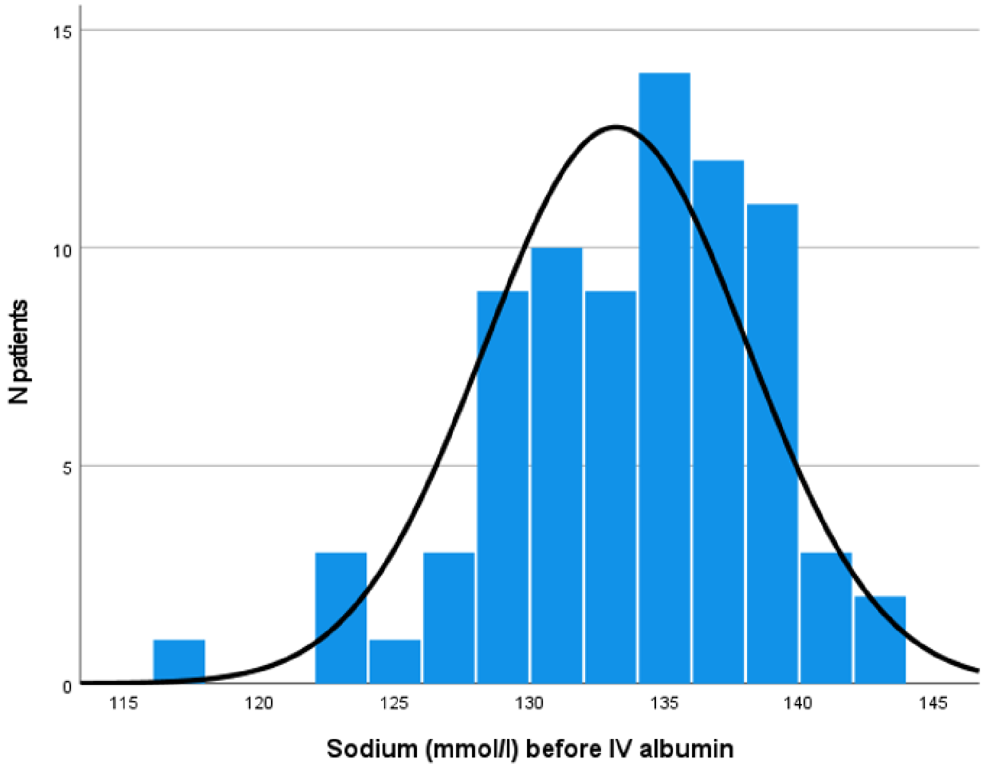

Abbreviations: IV: intravenous; N: number of patients

Table S1: Potential intravascular volume overload before and after passive leg raise and intravenous albumin

	All measurements (n= 81)		Individual patients (first inclusion, n= 55)	
**Potential severe intravascular volume overload (IVC^{max} >2.1 cm and IVCCI<20%)**	**Yes**	**No**	**Yes**	**No**
Volume overload at baseline	2 (2.5%)	79 (97.5%)	1 (1.8%)	54 (98.2%)
Volume overload at PLR	3 (3.7%)	78 (96.3%)	2 (3.6%)	53 (96.4%)
Volume overload before IV albumin	2 (2.5%)*	79 (97.5%)	1 (1.8%)	54 (98.2%)
Volume overload after IV albumin	17 (21.0%)*	64 (79.0%)	13 (23.6%)	42 (76.4%)
New volume overload after IV albumin	17 (21.0%)	64 (79.0%)	13 (23.6%)	42 (76.4%)
**Any potential intravascular volume overload (IVC^{max} >2.1 cm and IVCCI<50%)**	**Yes**	**No**	**Yes**	**No**
Volume overload at baseline	5 (6.2%)	76 (93.8%)	3 (5.5%)	52 (94.5%)
Volume overload at PLR	10 (12.3%)	71 (87.7%)	7 (12.7%)	48 (87.3%)
Volume overload before IV albumin	7 (8.6%)*	74 (91.4%)	3 (5.5%)	52 (94.5%)
Volume overload after IV albumin	42 (51.9%)*	39 (48.1%)	29 (52.7%)	26 (47.3%)
New volume overload after IV albumin	36 (44.4%)	45 (55.6%)	26 (47.3%)	29 (52.7%)

Legend: IV: intravenous, IVC^{max}: maximal diameter of the inferior vena cava, IVCCI: inferior vena cava collapsibility index, n: number of participants, PLR: passive leg raise

Table S2: Baseline characteristics stratified by presence of potential severe volume overload after IV albumin (all measurements n= 81)

	Potential severe intravascular volume overload (n=17)	No severe intravascular volume overload (n=64)	p-value
**Sex**			
Male (n, %)	8 (47.1%)	51 (79.7%)	**0.007**
Female (n, %)	9 (52.9%)	13 (20.3%)	
**Age (years, median, IQR)**	64 (51-68)	62 (57-68)	0.986
**BMI (kg/m^2, median, IQR)**	23.5 (21.9-25.1)	24.3 (21.4-28.2)	0.248
**Body surface area (m^2, median, IQR)**	1.75 (1.67-2.00)	1.90 (1.73-2.10)	0.091
**Child Pugh Score**			
B (n, %)	9 (52.9%)	38 (59.3%)	0.633
C (n, %)	8 (47.1%)	26 (40.7%)	
**MELD Score (median, IQR)**	17 (13-21)	14 (10-19)	0.094
**Creatinine (umol/l, median, IQR)**	92 (71-150)	93.5 (75.3-117.8)	0.472
**Sodium (mmol/l, median, IQR)**	136 (132-138)	133 (129-136)	**0.049**
**Potassium (mmol/l, median, IQR)**	4.0 (3.9-5.1)	4.1 (3.8-4.7)	0.288
**Diuretic use***			
Torasemide PO (mg, median IQR),	n=10, 10 (5-10)	n=43, 10 (10-20)	0.213
Spironolactone PO(mg, median, IQR),	n=10, 75 (43.8-200)	n=43, 100 (50-250)	0.182
Eplerenone PO (mg, median, IQR)	n=3, 50 (50-50)	n=6, 25 (25-57.5)	0.167
**Etiology of cirrhosis**			
ALD (n, %)	9 (52.9%)	36 (56.3%)	0.199
MetALD (n, %)	3 (17.6%)	15 (23.4%)	
MASH (n, %)	2 (11.8%)	6 (9.4%)	
Miscellaneous (n, %)	3 (17.6%)	7 (10.6%)	
**Presence of any varices**	10 (62.5%)	37 (58.7%)	0.784
**Presence of high-risk varices**	10 (62.5%)	33 (51.6%)	0.433
**Indication for IV albumin infusion**			
Large volume paracentesis (n, %)	16 (94.1%)	59 (92.2%)	0.787
Acute kidney injury (n, %)	3 (17.6%)	8 (12.5%)	0.582
**IV albumin dosage (g, median, IQR)**	40 (20-40)	40 (20-60)	0.261
**MAP measurements**			
MAP before IV albumin (mmHg, median, IQR)	n=17, 74.3 (64.5-78.3)	n=60, 82.3 (73.8-90.2)	**0.006**
MAP during IV albumin (mmHg, median, IQR)	n=17, 68.7 (64-74.4)	n=57, 77.3 (70.5-84.0)	**0.001**
MAP after IV albumin (mmHg, median, IQR)	n= 17, 68.3 (63.3-75.7)	n=57, 72.3 (67.8-84.3)	**0.033**
**Quantity of ascites removed (l, median, IQR)**	5.6 (3.6-7.2)	5.4 (3.8-8.4)	0.804
**NT-proBNP levels**			
NT-proBNP before IV albumin	779 (378-1299)	438 (232-884)	0.288
NT-proBNP after IV albumin	919 (455-1914)	632 (331-1415)	0.475

Legend: ALD: alcoholic liver disease, BMI: body mass index, g: gram, IQR: interquartile range, IV: intravenous, l: liter, MAP: mean arterial pressure, MASH: metabolic-dysfunction associated steatohepatitis, n: number of participants, MELD: model for end-stage liver disease, MetALD: metabolic dysfunction and alcohol-related liver disease, , n: number of participants PO: per oral, * only a small proportion of furosemide IV and PO (n=3) in the entire cohort, therefore no median and IQR calculated.

Note: p-value ≤ 0.05 was considered statistically significant. All continuous variables with skewed distribution shown as median and interquartile range. Differences between groups analyzed using the Wilcoxon-Mann-Whitney U test. Categorical variables shown as absolute values and percentages. Differences between groups assessed by Fisher´s exact or chi-squared (χ2) tests, as appropriate.
