# Supplementary material for: Point-of-care ultrasound of the inferior vena cava for intravascular volume assessment during intravenous albumin infusion in patients with cirrhosis
Source: JHEP Rep. 2025 Aug 20;7(11):101559. doi: 10.1016/j.jhepr.2025.101559 (PMC12529371; doi:10.1016/j.jhepr.2025.101559)
Supplement: Multimedia component 4 [file mmc4.pdf]

Point-of-care ultrasound of the inferior vena cava for intravascular volume assessment during intravenous albumin infusion in patients with cirrhosis

Authors

Daniel Segna, Fabio Brazerol, Pompilia Radu, Gerard Angeles Fite, Jaime Bosch, Annalisa Berzigotti

Correspondence

daniel.segna@insel.ch (D. Segna).

Graphical abstract

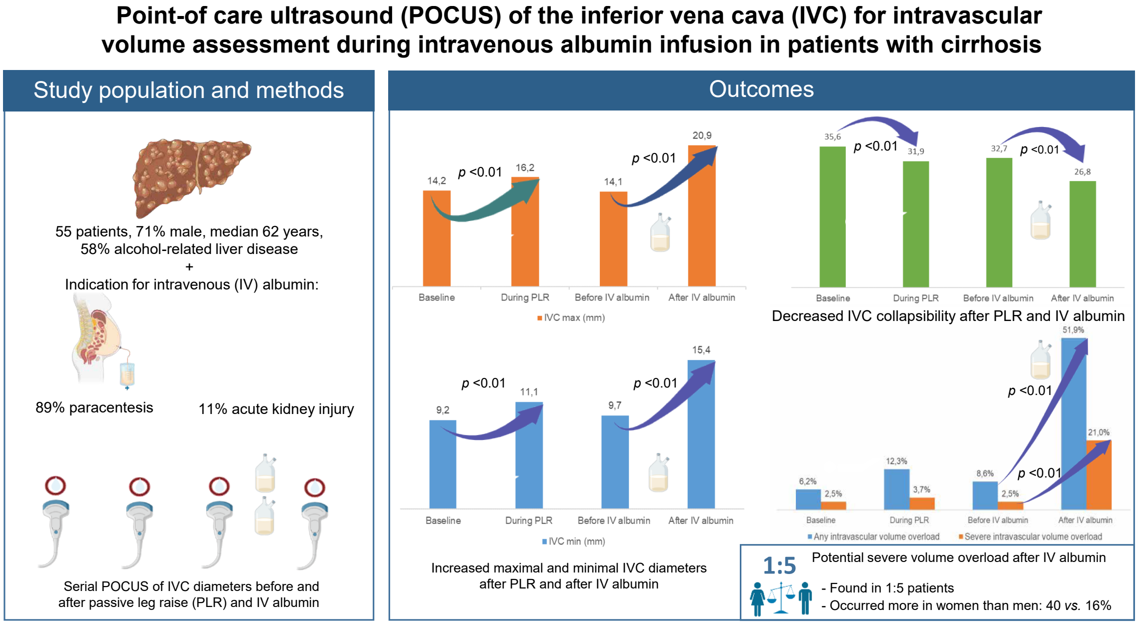

Highlights:

- IVC diameters increase after i.v. albumin, indicating increased intravascular volume.
- Potential severe volume overload occurred in 21% of patients after i.v. albumin.
- Potential severe volume overload affected more women than men after i.v. albumin.

Impact and implications:

Iatrogenic volume overload after albumin infusions in patients with cirrhosis is a potentially harmful side effect, but no policy for monitoring intravascular volume overload using non-invasive tools has been suggested so far. New-onset potential volume overload was detected in almost one out of five men and four out of 10 women and was associated with increases in N-terminal prohormone of brain natriuretic peptide, lower mean arterial pressure during albumin infusion, and higher serum sodium levels. Our results from this proof-of-concept study emphasize the need for larger prospective cohort studies to validate our findings and introduce strategies for individualizing volume management in patients with decompensated cirrhosis.

https://doi.org/10.1016/j.jhepr.2025.101559
© 2025 The Author(s). Published by Elsevier B.V. on behalf of European Association for the Study of the Liver (EASL). This is an open access article under the CC BY license (http://creativecommons.org/licenses/by/4.0/).

Point-of-care ultrasound of the inferior vena cava for intravascular volume assessment during intravenous albumin infusion in patients with cirrhosis

Daniel Segna[1,*], Fabio Brazerol[1], Pompilia Radu[1], Gerard Angeles Fite[1,2], Jaime Bosch[1,3], Annalisa Berzigotti[1,3]

JHEP Reports **2025**. vol. 7 | 1–9

Check for updates

Background & Aims: Current guidelines recommend intravenous (i.v.) albumin for different indications in decompensated cirrhosis, but iatrogenic hypervolemia following i.v. albumin is increasingly reported. We aimed to characterize intravascular volume status using point-of-care ultrasound (POCUS) of the inferior vena cava (IVC) during passive leg raise (PLR) and i.v. albumin, potentially facilitating clinical management and adjustment of albumin dosage.

Methods: This prospective pilot cohort included patients with decompensated cirrhosis requiring i.v. albumin. We assessed changes in minimal and maximal IVC diameters (IVC^{min} and IVC^{max}, respectively) and collapsibility index (IVCCI) during PLR and after i.v. albumin. We defined severe intravascular volume overload as IVC^{max} >2.1 cm and IVCCI <20%. Clinical outcomes were recorded until 3 months after POCUS.

Results: We included 81 measurements in 55 patients (70.9% men; median age 62 years; 58.2% alcohol-related cirrhosis; median Child-Pugh score 9 points; 89.1% paracentesis; median 40 g i.v. albumin; 5.5 L ascites). We found a significant increase in IVC diameters both during PLR (change in mean [Δ] IVC^{min} +20.7%, Δ IVC^{max} +14.1%, p <0.01) and after i.v. albumin (Δ IVC^{min} +58.8%, Δ IVC^{max} +48.2%, p <0.01). There was a significant decrease in IVCCI during PLR (relative Δ -11.1%, p <0.01) and after i.v. albumin (relative Δ -18.0%, p <0.01). Potential severe intravascular volume overload occurred on 17 occasions (21%) after i.v. albumin, more frequently in women than in men (40% *vs.* 15.7%, p <0.01), and showed higher cumulative incidence rates in variceal bleeding after 1 (16.7% *vs.* 0%, p = 0.01) and 3 months (18.2% *vs.* 0%, p = 0.01).

Conclusions: Potential severe intravascular volume overload after i.v. albumin was detected in every fifth patient with decompensated cirrhosis. Thus, there is a need to develop strategies for individualizing volume management in patients with decompensated cirrhosis.

© 2025 The Author(s). Published by Elsevier B.V. on behalf of European Association for the Study of the Liver (EASL). This is an open access article under the CC BY license (http://creativecommons.org/licenses/by/4.0/).

Introduction

Patients with cirrhosis and portal hypertension are prone to develop ascites. Current guidelines[1,2] recommend volume expansion using i.v. albumin infusion for several circumstances, such as large-volume paracentesis, spontaneous bacterial peritonitis (SBP), and treatment of acute kidney injury (AKI) with/without hepatorenal syndrome (AKI-HRS). In addition, i.v. albumin infusion has been advocated for diuretic-intractable ascites and for the prevention of recurrent ascites. However, current dosages for i.v. albumin infusion do not consider individual intravascular volume status before its administration. In addition, there are increasing reports of volume overload following volume expansion with albumin in patients with cirrhosis within[3] and beyond current indications.[4–6]

Non-invasive point-of-care ultrasound (POCUS) of the inferior vena cava (IVC) diameter and collapsibility index (IVCCI) using ultrasound has been proposed for assessing volume status in patients with cardiac disease[7] and for predicting fluid responsiveness in both mechanically ventilated and spontaneously breathing critically ill patients.[8] The passive leg raising (PLR) test produces an increase in venous return that mimics a fluid challenge and allows prediction of fluid responsiveness before volume infusion. Furthermore, it is not based on respiratory variations of stroke volume and, thus, remains reliable in patients breathing spontaneously.[9,10] In a meta-analysis, Monnet *et al.* reported an AUROC of 0.95 ± 0.01 for predicting fluid responsiveness changes provoked by PLR.[11] These non-invasive parameters also show a good accuracy for diagnosing volume overload in patients ventilated at low tidal volumes in intensive care units.[12] N-terminal prohormone of brain natriuretic peptide (NT-proBNP) is a biomarker recommended for the assessment of volume overload in the setting of heart insufficiency. Its short half-life makes it suitable for assessing changes in volume status

* Corresponding author. Address: Department for Visceral Surgery and Medicine, Inselspital Bern, Bern University Hospital, University of Bern, Freiburgstrasse 21, 3010 Bern, Switzerland. Tel.: +41-31-63-2-02-61.
E-mail address: daniel.segna@insel.ch (D. Segna).
https://doi.org/10.1016/j.jhepr.2025.101559

using serial measurements.[13] Moreover, increased left atrial volumes and elevated levels of NT-proBNP were significantly associated with severity of liver cirrhosis,[14] but changes in NT-proBNP have not been correlated with sonographic features of IVC as surrogate parameters for intravascular volume overload in patients with cirrhosis.

A proof-of-concept study assessed the role of IVC diameters and IVCCI in patients with cirrhosis scheduled for right heart catheterization. The authors concluded that IVC diameter and IVCCI accurately assess baseline intravascular volume status in patients with cirrhosis, potentially allowing for a non-invasive estimation of fluid requirements in this population.[15] However, there are no validated algorithms for guiding intravascular volume replacement using transabdominal ultrasound in patients with cirrhosis. This is largely because of the lack of data regarding the feasibility and response to physiological and pharmacological stimuli of POCUS of the IVC in the specific setting of decompensated cirrhosis. Thus, our study aimed to provide such evidence.

Patients and methods

Hypothesis and design

This was an exploratory single-center prospective cohort study assessing the impact of PLR and i.v. albumin infusion during standard medical treatment in patients with decompensated cirrhosis according to current indications for i.v. albumin. Data were recorded in an observational setting during standard medical treatment from October 2022 to October 2024 in outpatients and hospitalized patients at the Department of Visceral Surgery and Medicine, Bern University Hospital, Bern, Switzerland. This study was approved by the Cantonal Ethics Commission (BASEC: 2022-01226).

We hypothesized that changes in minimal and maximal IVC diameters and IVCCI (during the respiratory cycle) measured by transabdominal ultrasound increase during PLR and during i.v. albumin infusion in patients with cirrhosis, and that PLR changes might predict changes occurring during i.v. albumin infusion and with the occurrence of severe volume overload. We also hypothesized that changes in NT-proBNP would mirror changes in intravascular volume status.

Study population

We included ambulatory and hospitalized patients aged ≥18 years with liver cirrhosis of any etiology upon written informed consent. Diagnosis of cirrhosis was based on a combination of either histological, biochemical, radiological, and/or clinical parameters. We only considered patients with an indication for i.v. albumin infusion according to current EASL guidelines[1] and Baveno VII consensus recommendations,[2] including therapeutic paracentesis, spontaneous bacterial peritonitis (SBP), and/or AKI with/without AKI-HRS. No patients had a history of right heart failure or clinical evidence of lung edema, or recent hemodynamic instability/shock and all had had a transthoracic echocardiography within a maximum of 3 months of study inclusion.

Patients admitted to intermediate or intensive care units at the time of albumin infusion, with either recent i.v. albumin infusion within the previous 10 days, contraindications to PLR (*i.e.* increased intracranial pressure) or i.v. albumin (*i.e.* anaphylactic reactions), congenital valvulopathies, anatomic IVC abnormalities (*i.e.* IVC stenosis and/or thrombosis), or history of orthotopic liver transplant, and pregnant women were deemed not eligible.

Outcomes

Primary outcomes

Our primary outcomes were maximal and minimal IVC diameters (IVC^{max} and IVC^{min}, respectively), as well as IVC collapsibility indices (IVCCI) during a normal respiratory cycle without increased inspiratory effort (sniff maneuver).

All parameters were measured using POCUS at following time points: (1) before PLR, (2) during sustained PLR for 1 min,[16] (3) immediately before, and (4) after i.v. albumin infusion. IVCCI was calculated using the formula: $IVCCI = (IVC^{max} - IVC^{min})/IVC^{max}$.

Secondary outcomes

The first secondary outcome was prevalence and incidence of any potential and severe intravascular volume overload before and at PLR, as well as before and after i.v. albumin infusion. We used a broad definition for any potential intravascular volume overload combining IVC^{max} >2.1 cm, and IVCCI <50% according to Kaptein *et al.*[17] and the American guidelines for echocardiography of the right heart.[7] We defined severe volume overload using stricter criteria combining IVC^{max} >2.1 cm with IVCCI <20%, as suggested by Kaptein *et al.* in a recent study assessing the percentage of AKI-HRS misdiagnosis in decompensated cirrhosis.[17–19]

The second secondary outcome was the association between absolute values and changes in IVC^{max}, IVC^{min}, IVCCI, and NT-proBNP as surrogate parameters for potential intravascular volume overload in hospitalized patients. NT-ProBNP was measured the same day of i.v. albumin therapy and the subsequent day.

The third secondary outcome was cumulative incidence rate of clinical outcomes, such as death, recurrent paracentesis, AKI, AKI-HRS, hepatic encephalopathy (HE), SBP, and variceal bleeding at 1 and 3 months after baseline, stratified by potential severe intravascular volume overload.

The fourth outcome was correlation between NT-proBNP (in %) with IVC^{max}, IVC^{min}, and IVCCI (in %) and its changes during PLR and after i.v. albumin infusion. Assessing changes in IVC diameters and IVCCI in relation to dynamics in NT-proBNP helps elucidate the reliability of POCUS compared with an established blood-based biomarker for intravascular volume overload.

The final outcome was the correlation between changes in IVC parameters resulting from PLR and post-i.v. albumin infusion.

A direct correlation between changes in IVC diameters and IVCCI provoked by PLR with the same variables after i.v. albumin infusion will help collect the first data on the predictive ability of PLR regarding impending volume overload and fluid responsiveness in patients with decompensated cirrhosis.

Measurements and data extraction

Point-of-care ultrasound

POCUS was performed using any of three ultrasound devices (ARIETTA 65, FUJIFILM Healthcare Europe, Düsseldorf,

Germany; Philips Affiniti 50G – ultrasound system, Amsterdam, Netherlands; and GE Healthcare VenueFit^{TM}, Chicago, Illinois, United States of America) in outpatients and hospitalized patients at the Department of Visceral Surgery and Medicine and all images/sequences were documented in a centrally coded register for the sake of internal review for accuracy and reproducibility. Two independent operators with at least 5 years of experience in abdominal sonography performed POCUS.

The IVC diameters were measured 1–2 cm distally to the hepatic vein inlet into the IVC on spontaneous breathing with a convex probe in B-mode using either the subcostal long-axis window or mid-axillary window in case of obesity/surgery or intestinal air interposition.[17] PLR was the first step during the assessment. Based on Monnet *et al.*,[16] the first sequence of IVC^{max} and IVC^{min} during the respiration cycle was performed in a semi-recumbent position (45° head elevation) with compression stockings removed if present. Subsequently, the lower limbs were raised to an angle of 45° with the trunk in a completely supine position (guaranteeing a constant angle of 135° between lower extremities and the trunk). The second sequence of IVC diameters during the respiration cycle was measured 1 min after sustained PLR.[9] After repositioning of the patient in a semi-supine position from 0 to 45° head-of-bed elevation, IVC^{max} and IVC^{min} during the entire respiratory cycle were recorded immediately before and after i.v. albumin. The latter was administered at a maximum infusion rate of 20 g/h. In cases of paracentesis, POCUS was performed immediately before the intervention and, on average, 60 min after the final i.v. albumin infusion and drainage withdrawal. During paracentesis, only a minimal amount of intravenous crystalloids was infused for the permeability of the peripheral venous access, most commonly NaCl 0.9% (median 250 ml/24 h, IQR 100–500 ml/24 h).

Clinical information at baseline

The following biochemical variables were recorded at baseline: age (years), date of birth, sex, weight, height, BMI, etiology of liver cirrhosis, Child-Pugh-Turcotte score, Model for End-Stage Liver disease score (MELD), serum creatinine and sodium at baseline, and NT-proBNP serum levels the same and subsequent day of i.v. albumin infusion. Mean arterial pressure (MAP) was calculated from systolic and diastolic blood pressure measurements before, during, and after i.v. albumin.

Clinical outcomes

Patients were followed-up for 3 months after study inclusion and occurrence of AKI, AKI-HRS, SBP, HE, variceal bleed, and recurrent ascites was documented with the first date of incidence. Clinical information was retrieved from internal and external patient information systems or by direct contact with general practitioners or other specialists.

Data analysis

All analyses were performed in SPSS Statistics (package version 25). A two-sided p value ≤0.05 was considered statistically significant in all analyses. Continuous variables were presented as mean ± SD or median and IQR, as appropriate. For data with a normal distribution, differences between groups were analyzed using a two-sample independent t test. Continuous variables with skewed distribution were shown as median and IQR, and differences between groups were analyzed using the Wilcoxon–Mann-Whitney U test. Categorical variables were shown as percentages and associations assessed by Fisher's exact or chi-squared tests.

We used two datasets for the analysis. The main analysis comprised all measurements, allowing for multiple inclusion in the study (n = 81). In sensitivity analyses, we used data from individual patients using the measurements for the first inclusion (n = 55).

Absolute and relative changes in IVC^{max}, IVC^{min}, and IVCCI before and 1 min after sustained PLR, as well as immediately before and after i.v. albumin infusion, were analyzed separately using one-sample t tests (two-sided). When directly comparing absolute and relative changes of IVC^{max}, IVC^{min}, and IVCCI before and 1 min after sustained PLR *vs.* before and after i.v. albumin infusion, paired t tests were performed.

When testing the influence of the change in IVC^{max}, IVC^{min}, and IVCCI during PLR on the change during i.v. albumin infusion, we used a multivariable, repeated-measures, mixed-effects regression model adjusting for potential confounders, such as age, sex, and BMI.

Changes in prevalence of potential intravascular volume overload before and at PLR, as well as before and after i.v. albumin infusion, were analyzed using the McNemar test for paired binary data. Associations between changes in NT-proBNP (before/the morning after POCUS) and absolute values and changes in IVC^{max}, IVC^{min}, and IVCCI (during PLR, and before and after i.v. albumin) were assessed using multivariable regression models for continuous parameters, adjusted for potential confounders, such as age, sex, BMI, and MELD score. Finally, we used multivariable linear regression models for the association between NT-proBNP and prevalence/incidence of potential intravascular volume overload adjusted for confounders, such as age, sex, BMI, MELD score, and creatinine, in a stepwise approach. We provided stratified analyses for severity of liver cirrhosis according to Child-Pugh-Turcotte stage (B *vs.* C), quantity of ascites removed (<5 L *vs.* ≥5 L) and sex (female *vs.* male).

For the assessment of intraobserver variability, we compared IVC^{min} and IVC^{max} before PLR and i.v. albumin infusion, because they reflect the same conditions, and calculated the coefficient of variation (CV = SD/mean) accordingly.

Results

We included 55 patients (70.9% men; median age, 62 years; 58.2% alcohol-related cirrhosis; 58.2% Child-Pugh stage B; median Child-Pugh score, 9 points; median MELD score, 15), who were studied on 81 occasions. The most common indication for i.v. albumin infusion (median 40 g) was paracentesis in 89.1% of patients (median 5.5 L ascites, Table 1). Of these patients, 14 were included more than once (median 2 inclusions, IQR 2–3). All POCUS parameters were measured in the entire cohort upon obtaining informed consent.

Table 1. Baseline characteristics.

Characteristics	All measurements (n = 81)	Individual patients (first inclusion, n = 55)
Sex		
Male, n (%)	59 (72.8)	39 (70.9)
Female, n (%)	22 (27.2)	16 (29.1)
Age (years, median, IQR)	62 (57–68)	62 (57–67)
BMI (kg/m^2, median, IQR)	24.2 (21.5–27.7)	24.2 (21.3–27.7)
Child-Pugh score		
B, n (%)	47 (58)	32 (58.2)
C, n (%)	34 (42)	23 (41.8)
MELD score (median, IQR)	14 (11–19)	15 (11–19)
Creatinine (µmol/L, median, IQR)	93 (72–131)	97 (82–139)
Sodium (mmol/L, median, IQR)	134 (130–137)	134 (130–137)
Potassium (mmol/L, median, IQR)	4.1 (3.8–4.8)	4.1 (3.7–4.9)
Diuretic use*		
Torasemide PO (mg, median IQR),	n = 53, 10 (7.5–20)	n = 31, 10 (5–15)
Spironolactone PO (mg, median, IQR)	n = 53, 100 (50–200)	n = 33, (62.5–200)
Eplerenone PO (mg, median, IQR)	n = 9, 100 (25–65)	n = 6 (25–57.5)
Etiology of cirrhosis		
ALD, n (%)	45 (55.6)	32 (58.2)
MetALD, n (%)	18 (22.2)	10 (18.2)
MASH, n (%)	8 (9.9)	4 (7.3)
Miscellaneous (n, %)	10 (12.3)	9 (16.3)
Indication for i.v. albumin infusion		
Large-volume paracentesis, n (%)	75 (92.6)	49 (89.1)
Acute kidney injury, n (%)	11 (13.6)	11 (20.0)
Dosage for albumin infusion (g, median, IQR)	40 (20–60)	40 (20–60)
Quantity of ascites removed (L, median, IQR)	5.5 (3.8–8.0)	5.5 (3.5–8.0)

ALD, alcoholic liver disease; MASH, metabolic dysfunction–associated steatohepatitis; MELD, model for end-stage liver disease; MetALD, metabolic dysfunction and alcohol-related liver disease; PO, per oral.
*Only a small proportion of furosemide i.v. and PO (n = 3).

Changes in IVC^{max}, IVC^{min}, and IVCCI after PLR and i.v. albumin infusion

In our cohort of 81 measurements (Table 2, Fig. 1), we found a significant increase in IVC^{min} and IVC^{max} both during PLR (mean IVC^{min} 9.2 to 11.1 mm [+20.7%], mean IVC^{max} 14.2 to 16.2 mm [+14.1%], all p <0.01) and after i.v. albumin (mean IVC^{min} 9.7 to 15.4 mm [+58.8%], mean IVC^{max} 14.1 to 20.9 mm [+48.2%], all p <0.01). Moreover, we observed a significant decrease in IVCCI during PLR (mean IVCCI 35.6 to 31.9%; relative % change: -11.1%, p <0.01) and after i.v. albumin (mean IVCCI 32.7 to 26.8%; relative % change: -18.0%, p <0.01) After i.v. albumin, changes in IVC^{min} and IVC^{max} were significantly higher and IVCCI significantly lower compared with respective changes during PLR**.** These results were similar in a sensitivity analysis only including data from 55 individual patients at their first inclusion (Table 2).

In multivariate regression models adjusting for age, sex, and BMI, changes in IVC^{min}, IVC^{max}, and IVCCI during PLR did not predict those after i.v. albumin. In terms of intraobserver variability, CV for IVC^{min} (median 5.9%, IQR 0.9–18.8%) and IVC^{max} (median 6.1%, IQR 1.5–16.3%) indicated a rather low intraobserver variability.

Potential intravascular volume overload after PLR and i.v. albumin infusion

Taking into account all performed measurements (n = 81), any degree of potential intravascular volume overload (IVC^{max} >2.1 cm, IVCCI <50%) was present at baseline in five patients (6.2%), and doubled during PLR (10 patients, 12.3%, p = 0.06) and was almost 9-fold higher after i.v. albumin (42 patients, 51.9%, p <0.01). Any potential new-onset intravascular volume overload after i.v. albumin occurred in 36 patients (44.4%, 38.9% of whom were women; median age, 65 years; median BMI, 24.2 kg/m^2; median Child-Pugh score, 9 points, median MELD, 16 points; indications: 94.4% paracentesis; median albumin infusion, 40 g, Table S1).

Two patients (2.5%) showed potential severe intravascular volume overload (IVC^{max} >2.1 cm, IVCCI <20%) at baseline. This increased to three patients (3.7%) after PLR (p = 0.06) and to 17 patients (21%, p <0.01) after i.v. albumin (Fig. 1; Table S1). The comparison of baseline characteristics between patients with and without potential severe intravascular volume overload after i.v. albumin showed a marked female preponderance, significantly lower MAP values, and higher sodium levels (Table S2). New-onset potential severe intravascular volume overload after i.v. albumin was also found on 17 occasions (21%, 52.9% of whom were women; median age, 64 years; median BMI, 23.5 kg/m^2; median Child-Pugh score, 9 points; median, MELD 17 points; indications: 82.3% paracentesis, 11.8% paracentesis and AKI >1b, AKI >1b 5.9%; median albumin infusion, 40 g); in two patients, IVC^{max} and IVCCI fluctuated around the threshold for potential severe intravascular volume overload at baseline and before i.v. albumin.

There was a trend for a higher prevalence for any potential intravascular volume overload in women *vs.* men (68.2% *vs.* 45.8%, p = 0.07) only after i.v. albumin infusion, whereas patients with <5 L of ascites removed showed a higher prevalence of any potential intravascular volume overload at PLR (20.5% *vs.* 2.3%, p = 0.01) and before i.v. albumin (17.6% *vs.* 2.4%, p = 0.02) than those with >5 L of ascites removed with no significantly different prevalence before PLR and after i.v.

Table 2. Changes in diameters and collapsibility of the IVC before and after PLR and i.v. albumin.

Metric	All measurements (n = 81)			Individual patients (first inclusion, n = 55)		
	Mean	SD	*p* value	Mean	SD	*p* value
IVC^{max} before PLR (mm)	14.2	4.3	—	14.2	4.3	—
IVC^{min} before PLR (mm)	9.2	3.9	—	9.0	3.9	—
IVCCI before PLR (%)	35.6	14.1	—	37.1	14.6	—
IVC^{max} during PLR (mm)	16.2	4.4	—	16.2	4.5	—
IVC^{min} during PLR (mm)	11.1	4.0	—	11.0	4.0	—
IVCCI during PLR (%)	31.9	12.5	—	32.4	12.7	—
IVC^{max} before i.v. albumin (mm)	14.1	4.3	—	14.0	4.0	—
IVC^{min} before i.v. albumin (mm)	9.7	4.3	—	9.4	4.1	—
IVCCI before i.v. albumin (%)	32.7	15.9	—	33.4	16.9	—
IVC^{max} after i.v. albumin (mm)	20.9	4.4	—	21.0	4.1	—
IVC^{min} after i.v. albumin (mm)	15.4	4.7	—	15.4	4.7	—
IVCCI after i.v. albumin (%)	26.8	13.4	—	27.8	14.0	—
Difference in IVC^{max} before and during PLR (mm)	2.0	1.9	<0.01	2.0	1.7	<0.01
Difference in IVC^{min} before and during PLR (mm)	1.8	1.7	<0.01	2.0	1.6	<0.01
Difference in IVCCI before and during PLR (%)	-3.7	11.2	<0.01	-4.7	11.8	<0.01
Difference in IVC^{max} before and after i.v. albumin (mm)	6.7	5.3	<0.01	7.0	4.7	<0.01
Difference in IVC^{min} before and after i.v. albumin (mm)	5.8	5.6	<0.01	6.0	5.3	<0.01
Difference in IVCCI before and after i.v. albumin (%)	- 5.9	18.5	<0.01	- 5.7	18.7	<0.01

Differences are statistically significant at *p* ≤0.05. Continuous variables presented as mean ± SD. Differences in IVC parameters between analyzed using one sample *t* tests (two-sided). IVC, inferior vena cava; IVCCI, inferior vena cava collapsibility index; IVC^{max}, maximal diameter of the inferior vena cava; IVC^{min}, minimal diameter of the inferior vena cava; PLR, passive leg raise.

albumin. Finally, there were no significant differences in any potential intravascular volume overload between patients with Child-Pugh B and C at any time point.

Women showed significantly higher proportions of potential severe intravascular volume overload after i.v. albumin compared with men (40% *vs.* 15.7%, *p* <0.01). In binomial logistic regression models, neither BMI, body weight, height, or body surface weakened this association. There was no difference in prevalence of potential severe intravascular volume overload in patients with Child-Pugh B *vs.* C or in patients with <5 L *vs.* ≥5 L of ascites removed at any time point.

Individual patients on first inclusion (n = 55)

There was a significant increase in the prevalence of any potential or severe intravascular volume overload before and after i.v. albumin infusion (*p* = 0.02 and *p* <0.01, respectively; Table S1). No significant sex-specific differences for any volume overload were identified. There was no significant difference in prevalence of any volume overload at any time point between Child-Pugh B and C patients.

Stratified analyses for changes in IVC^{max}, IVC^{min}, and IVCCI after PLR and i.v. albumin infusion

Please refer to supplementary analyses and Table 3.

Association between changes in NT-proBNP and changes in IVC^{max}, IVC^{min}, and IVCCI and potential intravascular volume overload after PLR and i.v. albumin infusion

Serial NT-proBNP measurements were obtained from 30 hospitalized patients at baseline and 1 day after i.v. albumin infusion (Fig. 2). In multivariable regression models adjusting for age, sex, BMI, and MELD, there was no significant association between changes in IVC^{max}, IVC^{min}, or VCCI after PLR and i.v. albumin or changes in NT-proBNP from baseline. Similarly, no association between changes in NT-proBNP and absolute values of IVC^{max}, IVC^{min}, and IVCCI was identified.

However, increasing NT-proBNP levels from baseline were significantly associated with potential severe intravascular volume overload after i.v. albumin in multivariable regression models adjusting for age, sex, BMI, and MELD (beta coefficient [β] = -0.433, *p* = 0.048). This association remained significant after further adjustment for creatinine (β = -0.482, *p* = 0.034). Any degree of potential intravascular volume overload using broader criteria was not associated with changes in NT-proBNP in any model.

In subgroups according to quantity of ascites removed (<5 L *vs.* ≥5 L), sex (men *vs.* women), and Child-Pugh-Turcotte score, (B *vs.* C), there was a significant association between potential severe intravascular volume overload after i.v. albumin and NT-proBNP in those patients with ≥5 L of ascites removed in multivariable models adjusting for age, sex, BMI, and MELD score (β = 0.972, *p* = 0.047).

Cumulative incidence of clinical outcomes and potential intravascular volume overload after i.v. albumin infusion and PLR

There was an increased cumulative incidence rate of variceal bleeding (*p* <0.01) both at 1 (16.7% *vs.* 0%, *p* = 0.01) and 3 months (18.2% *vs.* 0%, *p* = 0.01) from baseline in patients with potential severe volume overload after i.v. albumin (Table 4) compared with their respective controls. However, there was no significant difference in the incidence of recurrent ascites, mortality, AKI, AKI-HRS, SBP, and HE either at 1 or 3 months from baseline, and no significant difference in events in patients with any degree of potential intravascular volume overload (IVC^{max} >2.1 cm, IVCCI <50%)

There was no difference in the cumulative incidence of any outcome according to the presence of any potential intravascular volume overload after PLR. MAP measurements before,

Table 3. Changes in diameters and collapsibility of the IVC before and after PLR and i.v. albumin stratified for sex, quantity of ascites removed, and Child-Pugh-Turcotte stage.

Characteristic	Male (n = 59)		Female (n = 22)		<5-L ascites (n = 34)		≥5-L ascites (n = 42)		Child-Pugh B (n = 46)		Child-Pugh C (n = 35)	
	Mean (SD)	*p* value	Mean (SD)	*p* value	Mean (SD)	*p* value	Mean (SD)	*p* value	Mean (SD)	*p* value	Mean (SD)	*p* value
IVC^{max} before PLR (mm)	14.2 (4.0)	—	14.2 (5.1)	—	15.2 (5.0)	—	13.3 (3.4)	—	13.7 (4.0)	—	14.9	—
IVC^{min} before PLR (mm)	9.2 (3.7)	—	9.3 (4.5)	—	10.2 (4.4)	—	8.4 (3.3)	—	9.0 (3.9)	—	9.6	—
IVCCI before PLR (%)	35.4 (14.3)	—	36.0 (13.8)	—	34.0 (12.0)	—	35.4 (15.1)	—	35.0 (14.9)	—	36.46	—
IVC^{max} during PLR (mm)	16.1 (4.1)	—	16.3 (5.1)	—	17.0 (5.1)	—	15.1 (3.4)	—	15.7 (3.9)	—	16.9	—
IVC^{min} during PLR (mm)	11.0 (3.7)	—	11.3 (4.8)	—	12.3 (4.2)	—	10.0 (3.4)	—	10.6 (3.9)	—	11.6	—
IVCCI during PLR (%)	32.1 (11.6)	—	31.5 (14.8)	—	27.8 (10.4)	—	34.3 (13.0)	—	32.7 (12.8)	—	31.27	—
IVC^{max} before i.v. albumin (mm)	14.2 (4.2)	—	14.1 (4.6)	—	14.7 (5.1)	—	13.4 (3.6)	—	13.9 (4.3)	—	14.6	—
IVC^{min} before i.v. albumin (mm)	9.6 (3.9)	—	9.9 (5.2)	—	10.4 (4.6)	—	8.9 (3.7)	—	9.5 (4.4)	—	9.8	—
IVCCI before i.v. albumin (%)	32.9 (14.6)	—	32.1 (19.4)	—	30.6 (12.7)	—	34.0 (17.0)	—	32.2 (17.0)	—	33.62	—
IVC^{max} after i.v. albumin (mm)	20.4 (4.3)	—	22.1 (4.6)	—	20.3 (4.8)	—	21.4 (4.3)	—	21.0 (4.3)	—	20.9	—
IVC^{min} after i.v. albumin (mm)	14.6 (4.3)	—	17.7 (5.1)	—	15.0 (5.3)	—	15.7 (4.3)	—	15.4 (4.7)	—	15.5	—
IVCCI after i.v. albumin (%)	28.9 (13.2)	—	21.2 (12.4)	—	27.4 (14.4)	—	26.6 (12.6)	—	27.3 (13.0)	—	26.3	—
Difference in IVC^{max} before and during PLR (mm)	1.9 (2.1)	<0.01	2.2 (1.3)	<0.01	1.8 (1.9)	<0.01	2.1 (1.9)	<0.01	2.0 (1.5)	<0.01	2.0 (2.3)	<0.01
Difference in IVC^{min} before and during PLR (mm)	1.8 (1.5)	<0.01	2.0 (1.9)	<0.01	2.0 (1.7)	<0.01	1.6 (1.6)	<0.01	1.7 (1.3)	<0.01	2.0 (2.0)	<0.01
Difference in IVCCI before and during PLR (%)	-3.3 (11.0)	<0.01	-4.5 (11.7)	<0.01	-6.2 (12.1)	<0.01	-1.1 (10.4)	<0.01	-2.3 (9.8)	<0.01	-5.2 (12.7)	<0.01
Difference in IVC^{max} before and after i.v. albumin (mm)	6.2 (5.3)	<0.01	8.1 (5.1)	<0.01	5.4 (4.3)	<0.01	8.0 (5.9)	<0.01	7.1 (5.4)	<0.01	6.3 (5.3)	<0.01
Difference in IVC^{min} before and after i.v. albumin (mm)	5.0 (5.1)	<0.01	7.8 (6.5)	<0.01	4.5 (4.9)	<0.01	6.8 (6.0)	<0.01	5.9 (5.7)	<0.01	5.7 (5.6)	<0.01
Difference in IVCCI before and after i.v. albumin (%)	-4.0 (16.4)	<0.01	-11.0 (22.9)	<0.01	-3.1 (14.9)	<0.01	-7.4 (20.8)	<0.01	-4.9 (19.2)	<0.01	-7.3 (18.0)	<0.01

*Statistically significant difference between two groups at $p \leq 0.05$. Continuous variables presented as mean ± SD. Differences in IVC parameters analyzed using one -sample *t* tests (two-sided). IVC, inferior vena cava; IVCCI, inferior vena cava collapsibility index; IVC^{max}, maximal diameter of the inferior vena cava; IVC^{min}, minimal diameter of the inferior vena cava; PLR, passive leg raise.

during, and after i.v. albumin infusion were associated with potential severe intravascular volume overload in univariable regression analyses (β = -0.076, *p* = 0.006; β = -0.097, *p* = 0.004; β = -0.069, *p* = 0.045, respectively), while only MAP before and during i.v. albumin remained so in multivariable regression models adjusting for age and sex (β = -0.060, *p* = 0.046; β = -0.084, *p* = 0.017). By contrast, we did not find any association between MAP before, during, and after i.v. albumin with any potential intravascular volume overload post-infusion.

Discussion

To the best of our knowledge, this is the first study characterizing changes in intravascular volume status during i.v. albumin infusion in decompensated patients with cirrhosis using POCUS, mostly during paracentesis. POCUS of the IVC is a non-invasive, easily learnable, readily available, and cost-efficient technique, which is reflected by a feasibility rate of 100% in experienced hands in our study. It not only showed significant increases in IVC diameters and decreases in IVCCI after albumin infusion or PLR, as indicated by previous studies,[3,18] but also yielded clinically relevant and actionable findings.

Almost 20% of male and 40% of female participants in our main analysis presented *de novo* potential severe volume overload immediately after i.v. albumin therapy at the doses recommended by current guidelines, independently from differences in anthropometric characteristics, such as BMI and body surface area. Potential severe intravascular volume overload was associated with decreased MAP before and during i.v. albumin infusion and increased NT-proBNP levels compared with controls, pointing toward a potentially negative hemodynamic response with possible short-term changes in cardiac function. This raises concerns about potential overtreatment and emphasizes the need for reconsideration of current dosage recommendations for i.v. albumin therapy, especially in patients requiring therapeutic paracentesis. Based on our results, individualizing i.v. albumin dosages according to the POCUS-estimated baseline intravascular volume status and sex-specific aspects should be explored in properly designed future studies.

In patients presenting *de novo* potential severe intravascular volume overload after i.v. albumin, we also found higher cumulative incidence rates of variceal bleeding within 1 and 3 months from baseline; however, these results need further investigation in larger cohorts and during longer observation periods. These observations point toward a potential clinical relevance of short-term changes in POCUS measurements of IVC parameters after i.v. albumin and advocate further investigation.

Based on our results, post i.v. albumin POCUS could be used to define non-invasively potential intravascular volume overload in decompensated cirrhosis, thus filling a major gap in hepatology. We consider our results as representing a first step toward individualized volume management in patients with decompensated cirrhosis. If confirmed in independent cohorts, these results have the potential to reduce unnecessary and excessive doses of albumin infusion and its associated morbidity.

However, our pilot study did not confirm the hypothesis that PLR could be a valid challenge test for predicting the risk of potential intravascular volume overload and clinical outcomes

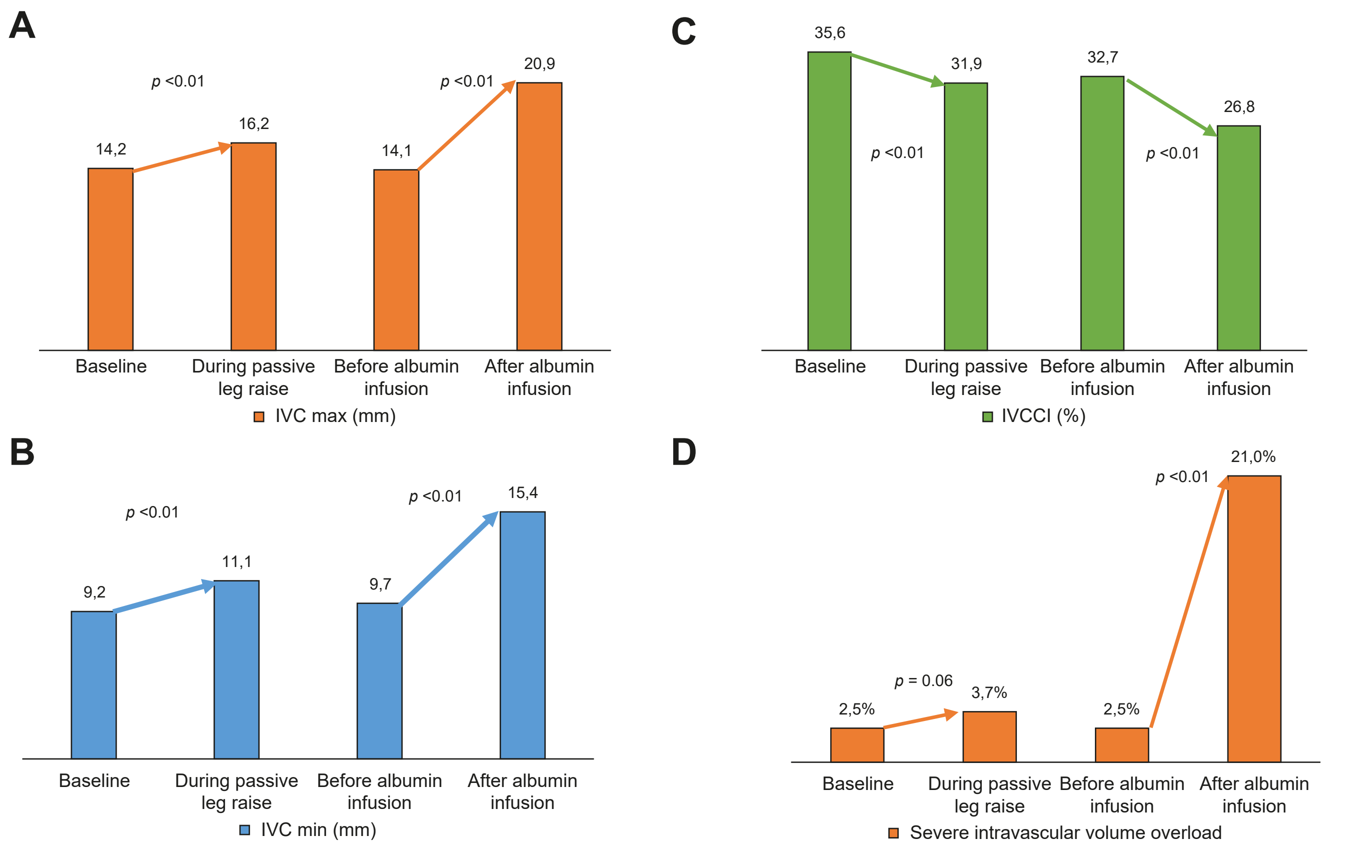

Fig. 1. Changes in mean IVC parameters and potential severe volume overload before and during passive leg raise, as well as before and after i.v. albumin infusion. (A) Changes in IVC^{max} (mm). (B) Changes in IVC^{min} (mm). (C) Changes in IVCCI (%). (D) Potential severe volume overload. Differences statistically significant at p <0.05. Differences in IVC parameters analyzed using one-sample t tests (two-sided). Differences in the prevalence of potential severe intravascular volume overload analyed using the McNemar test for paired binary data. IVC, inferior vena cava; IVC^{max}, maximum diameter of the inferior vena cava; IVC^{min}, minimum diameter of the inferior vena cava; IVCCI, inferior vena cava collapsibility index.

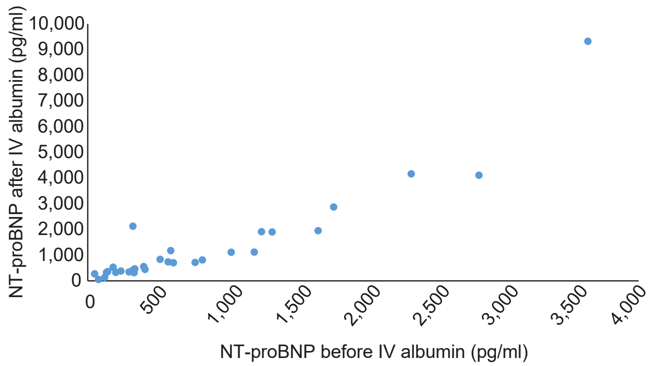

Fig. 2. Distribution of NT-proBNP in patients with decompensated liver cirrhosis before and after i.v. albumin. NT-proBNP, N-terminal pro brain natriuretic peptide.

after i.v. albumin infusions. We hypothesize that repeating POCUS at specified time points (50% and 75%) of the albumin infusion might give a more precise indication as to when to stop it if excessive increase in IVC^{max} and decrease in IVCCI are detected.

Our pilot study has some limitations to be addressed. First, POCUS is operator dependent and, as with any technique, it requires experience and adequate imaging circumstances. Given that most patients suffered from large-volume ascites and bloating, we chose a mid-axillary window for IVC measurements, which might generate slightly different values compared with a generally recommended subcostal approach. In addition, the assessment of intravascular volume status was non-invasive and, by study design, we were unable to compare POCUS findings with invasive parameters reflecting volume status. Nevertheless, this method is easily learnable and readily available in all kinds of medical facilities and should be seen as a screening method for prevalent or impending volume overload (or hypovolemia) in decompensated patients with cirrhosis.

Owing to the exploratory nature of the study, interobserver variability was not studied. Nevertheless, comparisons between repetitive measurements of IVC diameters before PLR and i.v. albumin yielded a low intraobserver variability, because they reflect the same situation. Future work could focus on this important aspect for ensuring applicability of this method. Second, cut-off values for defining volume overload according to IVC diameters and collapsibility vary across studies,[3,17–19] which led us to a dual analysis of strict and broad criteria defining potential severe and any intravascular volume overload, respectively. Only strict criteria were associated with changes in NT-proBNP and clinical outcomes, which emphasizes the need for a uniform definition of potential intravascular

Table 4. Distribution and cumulative incidence rate of clinical outcomes in potential severe intravascular volume overload after i.v. albumin.

Measurement	Severe intravascular volume overload after i.v. albumin	No severe intravascular volume overload after i.v. albumin	χ^2
**At 1 month (n = 50)**			
Recurrent ascites, n (%)			
Yes	8 (66.7)	16 (42.1)	0.138
No	4 (33.3)	22 (57.9)	
Variceal bleeding, n (%)			
Yes	2 (16.7)	0 (0)	0.010
No	10 (83.3)	38 (100)	
Death, n (%)			
Yes	0 (0)	6 (15.8)	0.142
No	12 (100)	32 (84.2)	
AKI, n (%)			
Yes	1 (8.3)	10 (26.3)	0.190
No	11 (91.7)	28 (73.7)	
AKI-HRS, n (%)			
Yes	0 (0)	3 (7.9)	0.315
No	12 (100)	35 (92.1)	
SBP, n (%)			
Yes	0 (0)	1 (2.6)	0.570
No	12 (100)	37 (97.4)	
HE, n (%)			
Yes	1 (8.3)	2 (5.3)	0.696
No	11 (91.7)	36 (94.7)	
**At 3 months (n = 45)**			
Recurrent ascites, n (%)			
Yes	8 (72.7)	19 (55.9)	0.322
No	3 (27.3)	15 (44.1)	
Variceal bleeding, n (%)			
Yes	2 (18.2)	0 (0)	0.011
No	9 (81.8)	34 (100)	
Death, n (%)			
Yes	1 (9.1)	7 (20.6)	0.386
No	10 (90.9)	27 (79.4)	
AKI, n (%)			
Yes	2 (18.2)	11 (32.4)	0.367
No	9 (81.8)	23 (67.6)	
AKI-HRS, n (%)			
Yes	1 (9.1)	2 (5.9)	0.711
No	10 (90.9)	32 (94.1)	
SBP, n (%)			
Yes	1 (9.1)	2 (5.9)	0.711
No	10 (90.9)	32 (94.1)	
HE, n (%)			
Yes	2 (18.2)	3 (8.8)	0.391
No	9 (81.8)	31 (91.2)	

Patients with IVC^{max} >2.1 cm and IVCCI <20%; individual patients with data from first inclusion. Differences are statistically significant at $p \leq 0.05$. Categorical variables shown as percentages and associations assessed by Fisher's exact or chi-squared ($\chi 2$) tests. AKI, acute kidney injury; AKI-HRS, acute kidney injury with hepatorenal syndrome; HE, hepatic encephalopathy; IVC^{max}, maximal diameter of inferior vena cava; SBP, spontaneous bacterial peritonitis.

volume overload in patients with decompensated cirrhosis with and without ascites.

Third, our study focuses on immediate changes in intravascular volume status after PLR and i.v. albumin without long-term serial POCUS measurements because of its exploratory nature. Although different models suggested an increased need for recurrent paracentesis and a decreased incidence of AKI during the first month on the one hand, and an increased incidence of variceal bleeding both at 1 and 3 months on the other hand, the long-term impact of these observations still needs to be elucidated in future work. This could help derive clinical algorithms for a tailored volume management and an individualized dosage of i.v. albumin according to sex, indication, and current intravascular volume status. Fourth, although a relevant increase in potential intravascular volume overload and a significant association with increased changes in NT-proBNP was noted, the latter can be significantly influenced by hepatic and renal function. Nevertheless, after correcting for the MELD score (which incorporates both factors), this association remained significant. Fifth, the study population included predominantly White participants with metabolic dysfunction and/or alcohol-related steatotic liver disease undergoing paracentesis. Therefore, our findings need to be confirmed for all i.v. albumin indications and other etiologies and ethnicities. Finally, IVC diameters and IVCCI in the abdominal region can be influenced by various degrees of intra-abdominal pressure before paracentesis (and i.v. albumin infusion).[20,21] Therefore, new-onset volume overload after i.v. albumin might be overestimated, and volume overload before i.v. albumin underestimated. Furthermore, the reliability of PLR might be reduced owing to a decreased venous return caused by elevated intra-abdominal pressure before paracentesis. However, stratified analyses for large-volume paracentesis *vs.* <5 L of ascites removed did not show different distributions of potential intravascular volume overload before and after i.v. albumin nor did they improve the prediction of volume overload after i.v. albumin using PLR.

There is limited literature on the role of POCUS of the IVC in volume management of patients with decompensated cirrhosis. Nevertheless, two cohort studies focused on the degree of misdiagnosis of AKI-HRS according to current guidelines in patients with decompensated cirrhosis with AKI and an i.v. albumin trial of minimum 48 h.[3,19] Both studies agree about a high degree of potential overdiagnosis of AKI-HRS and underline the importance of non-invasive assessments for diagnosing and managing potential intravascular volume overload in AKI in patients with decompensated cirrhosis. Our findings widen the spectrum of POCUS research in decompensated liver cirrhosis, given that our observations originate from the most frequent indication of i. v. albumin, namely counteracting circulatory dysfunction and volume depletion in patients undergoing therapeutic paracentesis. The findings indicate a potential for overtreatment with i.v. albumin in this population, especially in female patients.

In agreement with these studies, our findings corroborate the hypothesis that i.v. albumin therapy could be combined with at least baseline assessments of intravascular volume status using POCUS of the IVC. Moreover, serial POCUS measurements during volume therapy of any kind might be useful to monitor changes in intravascular volume status, as well as response to current volume management strategies. Future trials should validate our findings and should investigate short- and long-term effects of tailored concepts for i.v. albumin in larger cohorts to reduce potentially harmful and costly volume mismanagement in decompensated cirrhosis.

In conclusion, patients with decompensated cirrhosis showed significant increases in IVC diameters and decreases in IVCCI by POCUS not only after i.v. albumin, but also after PLR. Potential severe intravascular volume overload after i.v. albumin was detected in every fifth patient in general and in

40% of female patients. Our results emphasize that POCUS could be used in future studies to guide volume management and, in particular, to individualize i.v. albumin dosages in this fragile population.

Affiliations

[1]*Department of Visceral Surgery and Medicine, Inselspital, Bern University Hospital, University of Bern, Bern, Switzerland;* [2]*Department of Intensive Care Medicine, Inselspital, Bern University Hospital, University of Bern, Bern, Switzerland;* [3]*Department for BioMedical Research, Visceral Surgery and Medicine, University of Bern, Bern, Switzerland*

Abbreviations

AKI, acute kidney injury; AKI-HRS, hepatorenal syndrome; HE, hepatic encephalopathy; IVC, inferior vena cava; IVCCI, inferior vena cava collapsibility index; IVC^{max}, maximal diameter of the inferior vena cava during an entire respiratory cycle; IVC^{min}, minimal diameter of the inferior vena cava during an entire respiratory cycle; MAP, mean arterial pressure; MASH, metabolic dysfunction–associated steatohepatitis; MELD, model for end-stage liver disease; MetALD, metabolic dysfunction and alcohol-related liver disease; NT-proBNP, N-terminal prohormone of brain natriuretic peptide; PLR, passive leg raise; PO, per oral.; POCUS, point-of-care ultrasound; SBP, spontaneous bacterial peritonitis.

Financial support

The work was partially supported by a grant from the Berner Burgergemeinde (reference 2024-142). JB is supported by the Swiss Liver Foundation.

Conflicts of interest

DS reports travelling fees from Gilead and Falk. AB is a consultant for Boehringer Ingelheim and Astra Zeneca, and has received speakers' fees from GE Healthcare and Hologic. JB is a consultant for AstraZeneca, NovoNordisk, Boehringer Ingelheim, and Resolution Therapeutics. FB, GAF, and PS have nothing to disclose.

Please refer to the accompanying ICMJE disclosure forms for further details.

Authors' contributions

Study design: DS, AB, JB. Study set-up: DS, FB, AB, JB. Data collection and analysis: DS, FB, PR, GAF, AB. Manuscript writing: DS, AB, JB. Manuscript revision; DS, FB, PR, GAF, JB, AB. All authors approved the current version of the manuscript.

Data availability

All datasets generated and/or analyzed during this study can be obtained from the corresponding author upon reasonable request.

Acknowledgements

We would like to thank Lilian Smith and Judith Freiburghaus for their support throughout the duration of the project, and Andreas Limacher for his statistical feedback during the planning and analysis of this study.

Supplementary data

Supplementary data to this article can be found online at https://doi.org/10.1016/j.jhepr.2025.101559.

References

[1] EASL Clinical Practice Guidelines for the management of patients with decompensated cirrhosis. J Hepatol 2018;69:406–460.

[2] de Franchis R, Bosch J, Garcia-Tsao G, et al. B VII - renewing consensus in portal hypertension: report of the Baveno VII Consensus Workshop: personalized care in portal hypertension. J Hepatol 2022;76:959–974.

[3] Velez JCQ, Petkovich B, Karakala N, et al. Point-of-care echocardiography unveils misclassification of acute kidney injury as hepatorenal syndrome. Am J Nephrol 2019;50:204–211.

[4] Caraceni P, Riggio O, Angeli P, et al. Long-term albumin administration in decompensated cirrhosis (ANSWER): an open-label randomised trial. Lancet 2018;391:2417–2429.

[5] China L, Freemantle N, Forrest E, et al. A randomized trial of albumin infusions in hospitalized patients with cirrhosis. N Engl J Med 2021;384:808–817.

[6] Thévenot T, Bureau C, Oberti F, et al. Effect of albumin in cirrhotic patients with infection other than spontaneous bacterial peritonitis. A randomized trial. J Hepatol 2015;62:822–830.

[7] Rudski LG, Lai WW, Afilalo J, et al. Guidelines for the echocardiographic assessment of the right heart in adults: a report from the American Society of Echocardiography endorsed by the European Association of Echocardiography, a registered branch of the European Society of Cardiology, and the Canadian Society of Echocardiography. J Am Soc Echocardiogr 2010;23:685–713.

[8] Zhang Z, Xu X, Ye S, et al. Ultrasonographic measurement of the respiratory variation in the inferior vena cava diameter is predictive of fluid responsiveness in critically ill patients: systematic review and meta-analysis. Ultrasound Med Biol 2014;40:845–853.

[9] Monnet X, Teboul JL. Passive leg raising: five rules, not a drop of fluid. Crit Care 2015;19:18.

[10] Benomar B, Ouattara A, Estagnasie P, et al. Fluid responsiveness predicted by noninvasive bioreactance-based passive leg raise test. Intensive Care Med 2010;36:1875–1881.

[11] Monnet X, Marik P, Teboul JL. Passive leg raising for predicting fluid responsiveness: a systematic review and meta-analysis. Intensive Care Med 2016;42:1935–1947.

[12] Alvarado Sánchez JI, Caicedo Ruiz JD, Diaztagle Fernández JJ, et al. Predictors of fluid responsiveness in critically ill patients mechanically ventilated at low tidal volumes: systematic review and meta-analysis. Ann Intensive Care 2021;11:28.

[13] Mueller C, McDonald K, de Boer RA, et al. Heart Failure Association of the European Society of Cardiology practical guidance on the use of natriuretic peptide concentrations. Eur J Heart Fail 2019;21:715–731.

[14] Licata A, Corrao S, Petta S, et al. NT pro BNP plasma level and atrial volume are linked to the severity of liver cirrhosis. PLoS ONE 2013;8:e68364.

[15] Premkumar M, Rangegowda D, Kajal K, et al. Noninvasive estimation of intravascular volume status in cirrhosis by dynamic size and collapsibility indices of the inferior vena cava using bedside echocardiography. JGH Open 2019;3:322–328.

[16] Monnet X, Rienzo M, Osman D, et al. Passive leg raising predicts fluid responsiveness in the critically ill. Crit Care Med 2006;34:1402–1407.

[17] Kaptein MJ, Kaptein EM. Inferior vena cava collapsibility index: clinical validation and application for assessment of relative intravascular volume. Adv Chronic Kidney Dis 2021;28:218–226.

[18] Kaptein MJ, Kaptein JS, Oo Z, et al. Relationship of inferior vena cava collapsibility to ultrafiltration volume achieved in critically ill hemodialysis patients. Int J Nephrol Renovasc Dis 2018;11:195–209.

[19] Kaptein EM, Oo Z, Kaptein MJ. Hepatorenal syndrome misdiagnosis may be reduced using inferior vena cava ultrasound to assess intravascular volume and guide management. Ren Fail 2023;45:2185468.

[20] Nadim MK, Durand F, Kellum JA, et al. Management of the critically ill patient with cirrhosis: a multidisciplinary perspective. J Hepatol 2016;64:717–735.

[21] Nadim MK, Garcia-Tsao G. Acute kidney injury in patients with cirrhosis. N Engl J Med 2023;388:733–745.

Keywords: Point-of-care ultrasound; Inferior vena cava; Decompensated cirrhosis; Intravascular volume status.

Received 1 April 2025; received in revised form 5 August 2025; accepted 13 August 2025; Available online 20 August 2025

Journal of Hepatology, Volume *7*

Supplemental information

Point-of-care ultrasound of the inferior vena cava for intravascular volume assessment during intravenous albumin infusion in patients with cirrhosis

Daniel Segna, Fabio Brazerol, Pompilia Radu, Gerard Angeles Fite, Jaime Bosch, and Annalisa Berzigotti

Point-of-care ultrasound of the inferior vena cava for intravascular volume assessment during intravenous albumin infusion in patients with cirrhosis

Daniel Segna, Fabio Brazerol, Pompilia Radu, Gerard Angeles Fite, Jaime Bosch, Annalisa Berzigotti

Table of contents

Supplementary methods…………………………………………………………………2

Fig. S1…………………………………………………………………………………...3

Table S1………………………………………………………………………………4

Table S2………………………………………………………………………………5

Stratified analyses for changes in IVC^{max}, IVC^{min}, and IVCCI after PLR and IV albumin infusion

All measurements (n=81)

There were significant inter-sex differences with a significantly higher IVC^{min} and lower IVCCI after IV albumin in female vs. male participants (p=0.01 vs p= 0.02, respectively), whereas IVC^{max} was comparable between these two groups. In patients with more than 5 liters ascites removed and therefore undergoing large volume paracentesis (LVP), both IVC^{min} and IVC^{max} were significantly lower before PLR, whereas IVC^{min} was significantly lower and IVCCI significantly higher at PLR, compared to patients < 5 liters ascites removed. This effect was no longer present before and after IV albumin infusion. There were no significant differences between groups stratifying for Child Pugh Turcotte score (B vs C) at any time point (Table 3).

Individual patients with data from first inclusion (n=55)

In a sensitivity analysis only using data from the first inclusion of every patient, only IVCCI remained significantly lower in women than in men after IV albumin (21.8% vs 30.2%, p=0.04).In patients with LVP, IVC^{min} was significantly decreased (p=0.03) and IVCCI increased (p<0.01) at PLR, whereas IVC^{min} and IVCCI were significantly lower at PLR, compared to patients < 5 liters ascites removed. On the other hand, solely IVC^{max} was significantly higher in patients with LVP after IV albumin (22.5 vs 19.4 cm, p<0.01). When stratifying for Child stage, only IVC^{max} before PLR was significantly higher in Child C than B (15.7 vs. 13.1 cm, p=0.03) while all other parameters were not different in these two groups at any time point.

Fig. S1: Distribution of sodium (mmol) before IV albumin at baseline

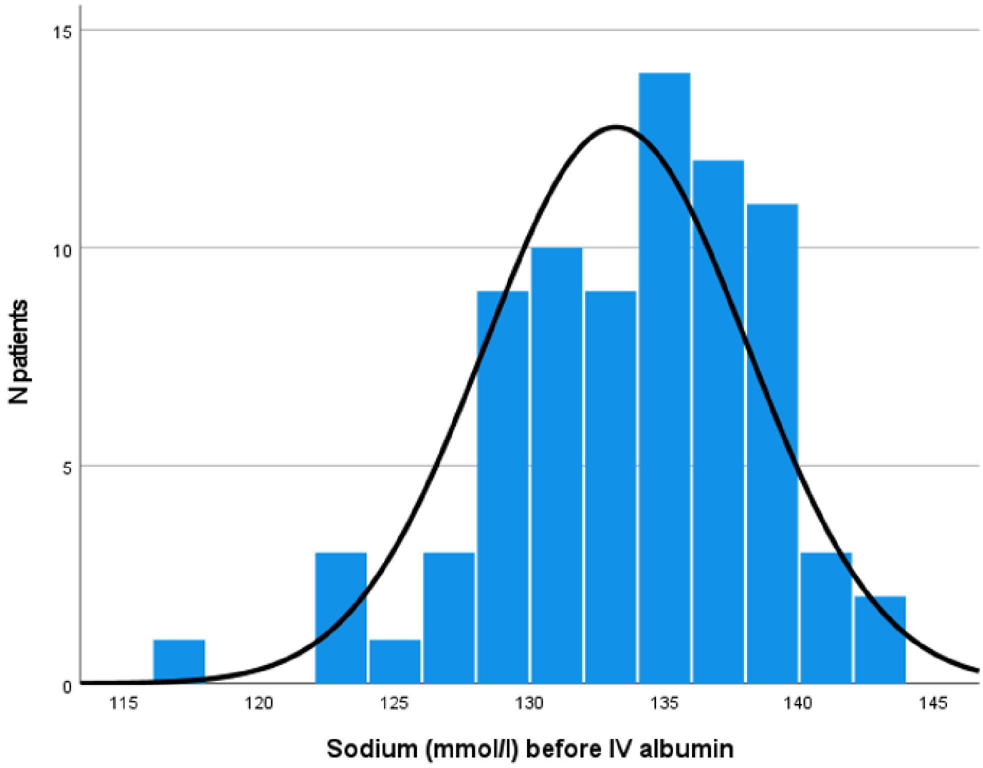

Abbreviations: IV: intravenous; N: number of patients

Table S1: Potential intravascular volume overload before and after passive leg raise and intravenous albumin

	All measurements (n= 81)		Individual patients (first inclusion, n= 55)	
**Potential severe intravascular volume overload (IVC^{max} >2.1 cm and IVCCI<20%)**	**Yes**	**No**	**Yes**	**No**
Volume overload at baseline	2 (2.5%)	79 (97.5%)	1 (1.8%)	54 (98.2%)
Volume overload at PLR	3 (3.7%)	78 (96.3%)	2 (3.6%)	53 (96.4%)
Volume overload before IV albumin	2 (2.5%)*	79 (97.5%)	1 (1.8%)	54 (98.2%)
Volume overload after IV albumin	17 (21.0%)*	64 (79.0%)	13 (23.6%)	42 (76.4%)
New volume overload after IV albumin	17 (21.0%)	64 (79.0%)	13 (23.6%)	42 (76.4%)
**Any potential intravascular volume overload (IVC^{max} >2.1 cm and IVCCI<50%)**	**Yes**	**No**	**Yes**	**No**
Volume overload at baseline	5 (6.2%)	76 (93.8%)	3 (5.5%)	52 (94.5%)
Volume overload at PLR	10 (12.3%)	71 (87.7%)	7 (12.7%)	48 (87.3%)
Volume overload before IV albumin	7 (8.6%)*	74 (91.4%)	3 (5.5%)	52 (94.5%)
Volume overload after IV albumin	42 (51.9%)*	39 (48.1%)	29 (52.7%)	26 (47.3%)
New volume overload after IV albumin	36 (44.4%)	45 (55.6%)	26 (47.3%)	29 (52.7%)

Legend: IV: intravenous, IVC^{max}: maximal diameter of the inferior vena cava, IVCCI: inferior vena cava collapsibility index, n: number of participants, PLR: passive leg raise

Table S2: Baseline characteristics stratified by presence of potential severe volume overload after IV albumin (all measurements n= 81)

	Potential severe intravascular volume overload (n=17)	No severe intravascular volume overload (n=64)	p-value
**Sex**			
Male (n, %)	8 (47.1%)	51 (79.7%)	**0.007**
Female (n, %)	9 (52.9%)	13 (20.3%)	
**Age (years, median, IQR)**	64 (51-68)	62 (57-68)	0.986
**BMI (kg/m², median, IQR)**	23.5 (21.9-25.1)	24.3 (21.4-28.2)	0.248
**Body surface area (m², median, IQR)**	1.75 (1.67-2.00)	1.90 (1.73-2.10)	0.091
**Child Pugh Score**			
B (n, %)	9 (52.9%)	38 (59.3%)	0.633
C (n, %)	8 (47.1%)	26 (40.7%)	
**MELD Score (median, IQR)**	17 (13-21)	14 (10-19)	0.094
**Creatinine (umol/l, median, IQR)**	92 (71-150)	93.5 (75.3-117.8)	0.472
**Sodium (mmol/l, median, IQR)**	136 (132-138)	133 (129-136)	**0.049**
**Potassium (mmol/l, median, IQR)**	4.0 (3.9-5.1)	4.1 (3.8-4.7)	0.288
**Diuretic use***			
Torasemide PO (mg, median IQR),	n=10, 10 (5-10)	n=43, 10 (10-20)	0.213
Spironolactone PO(mg, median, IQR),	n=10, 75 (43.8-200)	n=43, 100 (50-250)	0.182
Eplerenone PO (mg, median, IQR)	n=3, 50 (50-50)	n=6, 25 (25-57.5)	0.167
**Etiology of cirrhosis**			
ALD (n, %)	9 (52.9%)	36 (56.3%)	0.199
MetALD (n, %)	3 (17.6%)	15 (23.4%)	
MASH (n, %)	2 (11.8%)	6 (9.4%)	
Miscellaneous (n, %)	3 (17.6%)	7 (10.6%)	
**Presence of any varices**	10 (62.5%)	37 (58.7%)	0.784
**Presence of high-risk varices**	10 (62.5%)	33 (51.6%)	0.433
**Indication for IV albumin infusion**			
Large volume paracentesis (n, %)	16 (94.1%)	59 (92.2%)	0.787
Acute kidney injury (n, %)	3 (17.6%)	8 (12.5%)	0.582
**IV albumin dosage (g, median, IQR)**	40 (20-40)	40 (20-60)	0.261
**MAP measurements**			
MAP before IV albumin (mmHg, median, IQR)	n=17, 74.3 (64.5-78.3)	n=60, 82.3 (73.8-90.2)	**0.006**
MAP during IV albumin (mmHg, median, IQR)	n=17, 68.7 (64-74.4)	n=57, 77.3 (70.5-84.0)	**0.001**
MAP after IV albumin (mmHg, median, IQR)	n= 17, 68.3 (63.3-75.7)	n=57, 72.3 (67.8-84.3)	**0.033**
**Quantity of ascites removed (l, median, IQR)**	5.6 (3.6-7.2)	5.4 (3.8-8.4)	0.804
**NT-proBNP levels**			
NT-proBNP before IV albumin	779 (378-1299)	438 (232-884)	0.288
NT-proBNP after IV albumin	919 (455-1914)	632 (331-1415)	0.475

Legend: ALD: alcoholic liver disease, BMI: body mass index, g: gram, IQR: interquartile range, IV: intravenous, l: liter, MAP: mean arterial pressure, MASH: metabolic-dysfunction associated steatohepatitis, n: number of participants, MELD: model for end-stage liver disease, MetALD: metabolic dysfunction and alcohol-related liver disease, , n: number of participants PO: per oral, * only a small proportion of furosemide IV and PO (n=3) in the entire cohort, therefore no median and IQR calculated.

Note: p-value ≤ 0.05 was considered statistically significant. All continuous variables with skewed distribution shown as median and interquartile range. Differences between groups analyzed using the Wilcoxon-Mann-Whitney U test. Categorical variables shown as absolute values and percentages. Differences between groups assessed by Fisher´s exact or chi-squared (χ2) tests, as appropriate.
